# Medicated Inhalation

**Published:** 1855-11

**Authors:** 


### HALL'S JOURNAL OF HEALTH.

OUR LEGITIMATE SCOPE IS ALMOST BOUNDLESS: FOR WHATEVER BEGETS PLEASURABLE AND HARMLESS FEELINGS, PROMOTES HEALTH; AND WHATEVER INDUCES DISAGREEABLE SENSATIONS, ENGENDERS DISEASE.

VOL. II.] NOVEMBER, 1855. [NO. X.

### MEDICATED INHALATION.

WE have been repeatedly and urgently requested by the press and private persons, to notice some strictures, which we have neither seen nor read, on our July number, as to breathing the fumes of various medicines for curing consumptive diseases. We have asked no one what was the nature of the argument contained in these strictures, but we have understood from both the sources above named, that the article of "nine heavy columns" abounds in epithets and personalities against those who oppose the Inhalation treatment. Such being the state of the case, we now, as heretofore, avoid using names, and speak of the *Inhalists* as a class, and hereby take occasion not only to reiterate the two most important charges made in our previous articles, but give the recent testimony from the Inhalists themselves, in favor of the truth of our positions. First, that Medicated Inhalation for the treatment of consumption was no new thing; that it had been employed ages ago, had fallen into disuse, and now, for the dozenth time, was revived.

One of the Inhalists says, under date of October 13th of the present year, in the *New York Daily Times*,

"*All that I claim is, to have reduced the whole* (Medicated Inhalation) *to a systematized practice.*" If this is not abandoning the pretension to novelty and originality, in the employment of Medicated Inhalation, then are we ignorant of the meaning of language. Our first proposition is therefore admitted.

2. *That without other means, it was always and wholly inefficient to cure consumption.* As not one of these men at this time even professes to be confined to Medicated Inhalation exclusively in any case of consumption, but all use "*other means, according to*

*the case*," it is conclusive, that these "*other means*" must themselves be the principal agents, and 'Medicated Inhalation' becomes a mere *aid*, but as an "*aid*," it has been employed by regular physicians for the last hundred years. Our second proposition, therefore, requires no further proof.

3. We did admit that there might be some improvement in "*my* Inhaler," as it was so steadily termed, but from the published testimony of one of our oldest surgical instrument makers, we learn that he has sold them in this city since 1837, the only difference being, that "*my Inhaler*, CONTRIVED *by myself*," is in the shape of a bottle, the old ones, sold in New York "for the last *forty* years," being in the shape of a jar, the principle being identical, and as to construction, precisely similar, except in shape.

4. We protested against the Inhalists publishing the private letters of *inquiry* of physicians in different parts of the country, and heading that publication as *testimony of physicians in* FAVOR *of Inhalation.*

5. We protested against the statement of the Inhalists that they had thrown open certain instruments to the use of the profession, when these very instruments had been open to professional use by their real originators, for from ten to fifty years.

6. We protested against the Inhalists declaring that their motives in writing for the public press were to impart useful information to professional men, as well as the people, while, at the close of the same article, they give as a reason for not imparting such information to the profession, that they could not do so with "a proper regard for their own interests and reputation."

7. We protested against the validity of their reasons for not communicating their practice to the profession, that at its present stage, they could not make them understand all its niceties; that the knowledge of what remedy should be inhaled in any given case, or "how weak, or how strong, or how hot, or how cold, or how much, or how little, was only to be acquired by years of practical observation." We thought this an imputation on the capacity of medical men here and throughout the world for comprehension, as uncourteous in the extreme, believing as we do, that if a man knows anything wholly, he can communicate it to others of equal intelligence with himself.

8. We protested against the steadily repeated declarations of the Inhalists, that the object of their advertisements was to impart useful information on a class of diseases which destroys multitudes every year, when at the same time they have not, to this hour, imparted one single new truth in the whole sweep of medicine.

9. We protested against some of them, inasmuch as, being foreigners, and receiving largely of the patronage of our people, that they should subsidize the Sunday and penny press of New York almost without exception, and use it as a vehicle of defamation of the Medical profession of the United States, charging that the professors in our Medical Schools had purchased their professorships, that our physicians were "contracted in their views," were "ignorant," that they "knowingly" administered drugs which would do no good, and thereby "violated their own consciences."

We appeal to every well-regulated mind, if there is not exhibited here a want of courtesey, on the part of strangers who had been received with a bestowal of so large a share of public patronage, which was wholly uncalled for, because no public attack, either on the part of Medical professors or the physicians of the country, had been made against them, as far as we know.

10. We protested against the course pursued by "The American Medical Gazette and Journal of Health," in allowing Inhalists to advertise themselves in its pages for March, 1855; that in admitting an *Inhaler's* communication of eight pages, without a disclaimer, it used its influence in propagating the sentiments of the Inhalists, and thus caused the country journals of Medicine to believe that the *Gazette* was itself a believer in the claims of the Inhalists.

For this protest the "Gazette" has thought proper, in the October number, to class us with those who treat "diseases of horses and other cattle, Dr. Udolph Wolf (the celebrated vendor of German gin) and others." The severest reply which we propose making to this procedure, is the announcement that in this same number, the Gazette, for October, speaks of the men whom it helped into notice in March last, in the most contemptuous manner, declaring that they disclose nothing, because they have nothing to disclose; that the "*Specialist* has nothing novel or original on this (consumption) or any other subject, and that they, in common with other specialists, know less upon the subject which they have chosen, than their neighbors." In fact, the Gazette in effect, succinctly reiterates in its October issue, what we said in our July number. Why the Gazette should attempt severity towards us for doing what itself repeated some months subsequently, we pretend not to explain.

We take occasion here to state, for the information of the Gazette and others, that in all our writing, we have one aim distinctly in view—to say nothing which is not strictly and literally true. We pride ourselves in being true "with a margin," as a merchant would say. To be true, only with the help of a quibble, has always and everywhere our most unmitigated contempt. Our definition of a LIE is, *An attempt to make a false impression.* Whether successful or not, the crime is the same. Whether that false impression is made by word, or mark, or sign, or shrug, or look, or false coloring, whether by the omission or addition of a single jot or tittle, or word or fact, the infamy is the same. These being our sentiments, we have never undergone the humiliation of a retractation or apology for the defamation of any human creature, and that we have never been called to such a trial, and that no human heart has thus ever been wounded by us, is one of the sweetest thoughts of our existence. We are thankful to be able to feel, that we have no private malice to gratify, by using an editorial advantage, and making our Journal the vehicle of defamation of the character and standing and competency of those who, as private citizens and members of the profession, have for many years secured and maintained the respect and esteem not only of the private and professional community around them, but of men of ability and eminence in every part of the country.

What may take place in that cold domain on the *other* side of "Forty-Five," we cannot say. Into that hard and cheerless clime, the editor of the *Gazette* has long ago passed before us, will he telegraph us back whether, in that dreary land, age and frigidity are one and indivisible; if it be so, sad indeed will be the day when we shall become a citizen.

If we ever do, the very first thing we shall attempt, will be to publish a prospectus and canvass personally for subscribers to a "Journal of Reform;" meanwhile, we will strike for a revolution, and inaugurate an *Age of genial sunshine;* we will charter a *Caloric Company*, the object of which shall be to thaw every frozen heart, to melt down every face of brass, and warm beauteous flowers into life, where flowers never bloomed before.

No less than three thousand persons die of diseases of the lungs every year, in New York City alone; and in discussing the means of their cure, men want the argument of tangible facts, from known reliable witnesses. It is inopportune to attempt to divert public attention to side issues, to personalities, to abusive epithets, impugning motives, questioning veracity, and by the quibble of scrap quotations and want of fair construction, these being the weapons of the Inhalists against one another in almost every newspaper article we have read on the subject, and we have no reason to suppose they have used different weapons in articles which we have not seen against the medical profession in general or ourselves in particular. In a contest of this kind, we can never engage. Truth needs no help from sources like these: she can always afford to be courteous and calm, and borrows no aid from ribaldry or the stiletto.

The facts which we want as to the curative power of Inhalation over consumption, is the production of *one* intelligent and responsible citizen of New York who has been cured of this disease by Medicated Inhalation alone, of Consumption acknowledged to be present by any physician known to favorable fame, of any school. We do not want the affidavit of *John Smith*, "*his mark*," of New York; or of *Thomas Brown* of some distant locality: a million such are not worth a straw, and for very obvious reasons.

*Consumption* implies these four things mainly:

1. A pulse of ninety or more in a minute.
2. A breathing, at rest, of twenty-five or more in a minute.
3. A bad cough on going to bed, and on rising.
4. Great and increasing thinness of flesh.

We hereby challenge every Inhalist in the country, or anybody else, to produce one single person who presented such a condition at any past time, who *now*, through Inhalation alone,

1. Has a healthful pulse of sixty-eight.
2. A breathing of seventeen.
3. No remnant of cough.
4. No perceptible emaciation.

Surely, of the multitude of persons whom these men have treated, there ought to be found *one* well-known and intelligent man of high position, grateful enough for his deliverance, to be willing to bear an open and manly testimony to its healing power. There are multitudes of persons who have tried Medicated Inhalation in this city, and vainly tried it. Their disappointment has vented itself in various degrees of strength up to the point of declaring that "a more cruel imposition has never been practised on any community."

As a class, the Editors of this country are educated and cultivated men, and as such, are capable of appreciating the value of a moral argument, especially one which carries with it the force of a demonstration, and such an argument we conceive the following to be: Medicated Inhalation could not have become obsolete among a body of men as highly cultivated and scientific as the Medical Faculty throughout the world are, unless they had found that its employment was not followed by results sufficiently prompt, uniform, and effective, as to warrant its use, considering that the same ends were attained with greater facility, and certainty, by more available instrumentalities. They would no more abandon a signally useful remedy, than the Herald, Times, or Tribune, would abandon the six-cylinder press and go back to one of Franklin's time. There can be no motive for such a change. Medicines are like patent contrivances, they are laid aside or continued according to their availability.

The force of this argument will be met by the assertion, that these men have got up an "*Improved Inhalation*," some of them having used that phrase in their advertisements. The improvement must be in "*my instrument, contrived by myself*," or in the substances inhaled; but we have already shown that the Inhaler now is identically the same as it was "*forty years ago in New York*," excepting only, a jar was then used, while "*My Inhaler, contrived by myself*," is a bottle.

It cannot be the *substance* used, for they claim "the employment of the whole Materia Medica," "according to the nature of the case," while they themselves rest their professed success, to use their own language, in "knowing how much or how little, or how weak or how strong, or how hot or how cold the particular drug used should be," declining at the same time to communicate the necessary information as to these points on two grounds :—

First. It would compromise their *interest and reputation.*

Second. The Medical profession are incapable, at this stage of (their) discoveries, of understanding what remedy is most appropriate to any given case, or "how much or how little, or how weak or how strong, or how hot or how cold."

We need suggest no inferences here to any intelligent mind.

We are prepared to go a step farther, and say, "*Medicated Inhalation, alone, never cured any man, woman, or child, of anything, since the world began.*" I defy the universe to produce one solitary instance of such cure. The term "cured" is qualified thus: No disease, nor any one symptom of a disease, has ever been permanently removed by means of Medicated Inhalation alone, which disease or symptom has not, in multitudes of instances, disappeared by the most ordinary medical means, or even by simply being let alone. If such a thing has been done, let it be substantiated. Even admitting that Inhalation has cured somebody of something, if nature alone has done the same thing, and legitimate medicine has done the same thing, in untold instances, and this none of them will deny, and we take it for granted, until they name one symptom or one disease to the contrary, then the prime fact must force itself on every intelligent mind, that Medicated Inhalation is not entitled to preeminence as a means of cure, and that any attempt to produce an impression to the contrary will, sooner or later, receive the most general reprobation.

Another consideration, of no doubtful bearing, is, that in order to make the theory of Inhalation at all plausible, they contradict the entire medical world as to a point of fact. The very necessity of such a thing is sufficient to envelop the whole in suspicion. There is scarcely a single fact in the whole range of physiological medicine more deserving the title of "*fixed*" than that consumption is a constitutional disease, a disease of the whole man; and as an evidence, of popular appreciation, the whole man dwindles away, flesh, strength, breath, digestion, sleep, everything, yet these men begin by saying it is a local disease, that it is founded in the lungs; and with a view to cure the diseased portions, for it is only in spots or patches that the lungs waste away in consumption, they apply the same fumigations to the well parts as they do to the diseased, and what is potent to cure a diseased portion must be as powerful to injure the part which is in a healthy condition. Any well man may try this and breathe iodine or chlorine, the remedies which they most generally use, and see if cough and other unpleasant sensations do not instantaneously arise; keeping out of sight, as they do, the absurdity of curing an internal ulcer by means which, if used for one on the surface of the body, would expose any one to derision. We presume no one ever attempted to cure an external ulcer by blowing upon it the fumes of any drug. As well might you satisfy a hungry man with the smell of a good dinner, or a penniless unfortunate by the jingle of dollars.

We have no objection to a physician advertising himself, if he does it courteously towards his brethren and in all truthfulness; but to baseless pretensions, with defamation of others, we do enter an earnest protest.

As to our own mode of practice, confined to one kind of lung disease, that is, Consumption, and to one kind of throat disease, called Chronic Laryngitis, we say as to the latter, that in treating it as a dyspeptic disease, by orthodox medical principles, we have found our highest success; we utterly repudiate caustics in all their forms, believing them inert in some cases, hurtful in others, and curative in none.

As to Consumption, we have been satisfied to treat it as a constitutional disease, and think that in doing so, we have averted it in its forming stages, and arrested it, indefinitely, in the earlier stages of decay, the three great aims being—

1. The largest possible consumption of out-door air.
2. The largest possible amount of out-door exercise, short of fatigue.
3. The largest possible digestion of plain substantial food.

In treating these diseases thus, we are free to say, we have not, in some fifteen years practice in them, had that success which strikes a nation with surprise, and filled at one and the same time our office with patients, and our purse with "a hundred thousand dollars a year." We have made no efforts to lead our patients along by the aid of the mysterious and the wonderful; we have never said to any man, "We will certainly cure you—no doubt about it." The fact is, we never promised to cure anybody of any thing. We do, however, take pains, in all cases, to deal with our patients as rational beings, by patiently instructing them as to the nature of their ailments, the nature of their constitutions, their liabilities to disease, the principles by which we expect to cure them, and if cured, how they may keep well hereafter; and with the gratitude of many intelligent persons *and* their pay, as well as the approbation of my own conscience, I have great reason to be satisfied and thankful.

---

